# Solvent-Driven Enrichment and Multiplex Analysis of Local Anesthetics by Thin-Layer Chromatography Coupled with Surface-Enhanced Raman Spectroscopy

**DOI:** 10.3390/molecules30071585

**Published:** 2025-04-02

**Authors:** Huimin Sui, Miao Li, Yangyang Gao, Jie Luo, Fangyuan Ban, Tao Xu, Shuang Fu, Chao-Yang Zhao, Hailin Wen, Cuiyan Han

**Affiliations:** 1 School of Pharmacy, Qiqihar Medical University, Qiqihar 161006, China; suihm_9@163.com (H.S.); 19190706959@163.com (M.L.); 13283631961@163.com (Y.G.); 18074757884@163.com (J.L.); 18522488569@163.com (F.B.); harvey-333@163.com (T.X.); fsjt1980@163.com (S.F.); zhao33447@qmu.edu.cn (C.-Y.Z.); hlw395456@163.com (H.W.); 2 Postdoctoral Research Station, Qiqihar Institute of Medical and Pharmaceutical Sciences, Qiqihar 161006, China

**Keywords:** local anesthetics, TLC, SERS, multiplex analysis, Raman

## Abstract

Local anesthetics have been widely used in clinical analgesia due to their ability to provide effective regional pain management. Accurate measurement of local anesthetics in body fluids is crucial for ensuring patient medication safety and optimizing therapeutic efficacy. Herein, we present a convenient, economical, sensitive, and efficient TLC-SERS method for multiplex determination of six kinds of anesthetics (pro) in human plasma, including procaine hydrochloride (Pro), tetracaine hydrochloride (Tet), dibucaine (Dib), mepivacaine hydrochloride (Mep), lidocaine hydrochloride (Lid), and ropivacaine hydrochloride (Rop). The TLC method was adopted to separate six local anesthetics effectively. In order to improve the sensitivity, TLC spots were concentrated into smaller ones using methanol through solvent-driven enrichment, then Ag NPs staining was applied to enriched spots for a strong and unique SERS response of each anesthetic. As a result, linear calibration curves of SERS intensity ratio versus negative logarithm of spotting amounts sampled on TLC plates were obtained, along with the lowest detectable amounts in this study were 1 ng (Pro), 10 pg (Tet), 10 ng (Dib), 50 ng (Mep), 50 ng (Lid), and 0.1 μg (Rop), which were up to 2 × 10^4^ times more sensitive than our previous TLC-Raman method. Moreover, the method was successfully applied to human plasma samples, demonstrating the feasibility and potential for multiplex analysis of local anesthetics in clinical practice, criminal forensics, and aquaculture.

## 1. Introduction

Local anesthetics, such as lidocaine, procaine, and mepivacaine, are indispensable in medical practice for their ability to block nerve impulses and alleviate pain [1]. The issue of systemic toxicity associated with local anesthetics in clinical applications is becoming increasingly prominent. It occurs in approximately 1 out of every 1000 local anesthetic administrations, causing severe neurological and cardiovascular symptoms in patients, such as seizures, arrhythmias, cardiac arrest, and even death [2]. The use of local anesthetics involves various medical procedures, including anesthesia in operating rooms and local injections in outpatient settings. Differences in the technical proficiency and experience of operators may lead to misuse or overdose of local anesthetics, thereby increasing the risk of toxic reactions. Accurate and efficient detection of these drugs is crucial for ensuring patient safety and optimizing therapeutic outcomes. Despite significant advancements in the detection methods for local anesthetics, several challenges remain that hinder the widespread adoption and effectiveness of these techniques.

Mass spectrometry (MS) techniques, particularly liquid chromatography–tandem mass spectrometry (LC-MS/MS), have become powerful tools for detecting local anesthetics in plasma and aquatic products due to their exceptional sensitivity and specificity [3,4,5,6,7,8]. However, extensive and time-consuming sample preparation is often required, which can introduce variability and loss of analytes. Additionally, the high cost and the need for specialized operators restrict their practical use. Electrochemical methods have garnered significant attention due to their low cost, simplicity, and high sensitivity, but further studies should focus on improving their stability, specificity, and reproducibility [9,10,11,12,13,14,15,16]. Optical and spectroscopic methods [17,18,19], including UV-visible and fluorescence spectroscopy, offer non-invasive and real-time monitoring capabilities, making them valuable for clinical applications. Nevertheless, issues such as selectivity, high background noise, and matrix effects must be addressed. Therefore, more research is essential to address the remaining challenges and develop advanced methods that can simplify the sample treatment process, ensure high specificity and sensitivity, and enable safer and more effective use of local anesthetics in medical practice.

Surface-enhanced Raman spectroscopy (SERS) has emerged as a powerful analytical technique with remarkable potential in various scientific domains, owing to its exceptional sensitivity and molecular specificity. In biomedical applications, SERS has demonstrated significant capabilities in biomarker detection, early disease diagnosis, and drug screening [20]. SERS enables the detection of trace pollutants, including heavy metal ions and organic contaminants, offering a non-destructive and efficient method for environmental pollution assessment [21]. In addition, in the field of food safety, SERS has proven invaluable for rapid screening of pesticide residues, food additives, and various harmful substances, ensuring compliance with stringent food safety regulations [22]. The high-resolution capability of SERS at the molecular level has established it as an indispensable tool for monitoring chemical reactions [23], particularly in studying catalytic processes and elucidating reaction mechanisms. Consequently, SERS, with its outstanding characteristics of minimal sample preparation, low background interference, high sensitivity, and unique molecular fingerprint identification, stands out as a superior method for the detection of local anesthetics in both laboratory and field settings.

Our previous work has reported the determination of six caine-type anesthetics in cosmetics and rat serum by thin layer chromatography (TLC)-Raman spectroscopy with simple sample pretreatment, low cost, and good selectivity [24]. Although the sensitivity was improved compared to the TLC method, it was still insufficient for practical application. SERS has been widely used in various fields due to its extremely high sensitivity enabled by engineered nanomaterials such as gold, silver, and hybrid nanoparticles, which amplify Raman signals through plasmonic and chemical enhancement mechanisms. Building on this foundation, TLC-SERS has attracted substantial research interest by integrating separation and detection capabilities on an integrated platform, enabling simple and multiplex analysis of complex mixtures with preserved ultrahigh sensitivity. Therefore, Ag NPs, as a classic SERS substrate, were introduced into the detection system to achieve strong SERS signals of local anesthetics, including procaine hydrochloride (Pro), tetracaine hydrochloride (Tet), dibucaine (Dib), mepivacaine hydrochloride (Mep), lidocaine hydrochloride (Lid), and ropivacaine hydrochloride (Rop).

In this work, we describe a facile, selective, and sensitive TLC-SERS method for the multiplex analysis of local anesthetics through solvent-driven enrichment of TLC spots and localized surface plasmon resonance (LSPR) effect of Ag NPs. The target local anesthetics were separated by TLC, and the obtained TLC spots were concentrated into small ones driven by solvent using a capillary tube. The enriched spots were then immersed in Ag NPs colloidal solution and followed by SERS measurements. Improved sensitivity was achieved through the LSPR effect and solvent-driven enrichment. Furthermore, the feasibility of the TLC-SERS method was successfully verified by human plasma samples with simple sample pretreatment and experimental procedures, demonstrating definite potential for multiplex analysis of local anesthetics in practical applications.

## 2. Results and Discussion

### 2.1. TLC-SERS Strategy by Solvent-Driven Enrichment

**Figure 1:**
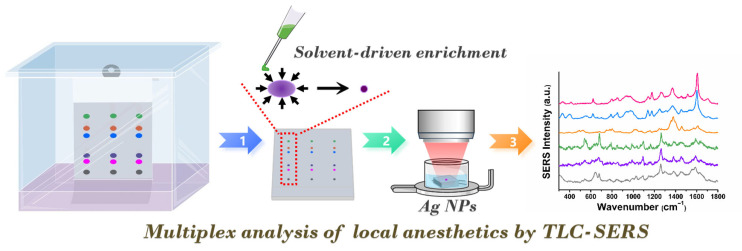
Solvent-driven TLC-SERS measurement of local anesthetics.

TLC is a classic and effective technique for the separation of substances with the merits of satisfactory separation efficiency, relatively rapid analysis, simple operation, and low cost. SERS is a facile and powerful analytical tool with high sensitivity and selectivity, enabling single-molecule detection and providing molecular fingerprint vibrational information. Herein, we proposed a simple, sensitive, selective, and effective TLC-SERS method for the multiplex determination of six types of anesthetics (Figure 1). The target anesthetics were effectively separated using TLC silica gel 60F_254_ with cyclohexane-triethylamine (13:7, *v*/*v*) as the developing solvent. The retention factor (*R*_f_) values were 0.08, 0.15, 0.40, 0.51, 0.60, and 0.73 for Pro, Tet, Dib, Mep, Lid, and Rop, respectively. The sample spots appear purple due to fluorescence quenching under a 254 nm ultraviolet lamp. Then, we enriched the TLC spots into small ones driven by solvent from different directions using a quantitative spotting capillary. Subsequently, the enriched sample spot was cut from the TLC plate, placed in the overturned cap of a 1.5 mL EP tube, and immersed in 10 μL of Ag NPs for subsequent in situ SERS determination under a 780 nm excitation wavelength. However, when the sample spotting amount is small, the color of the sample becomes invisible, which is not conducive to the subsequent concentration of spots. Since the *R*_f_ value of each anesthetic is constant under these experimental conditions, a corresponding standard solution with a high concentration was spotted on each TLC plate to enable spot enrichment based on the position of the standard solution. The distinct *R*_f_ values and inherent SERS vibrational information of the analytes provide the specificity required for multiplex analysis. Moreover, the SERS signals of local anesthetics enhanced by Ag NPs allow for highly sensitive detection. Therefore, the TLC-SERS strategy based on solvent-driven enrichment represents a potential tool for the simultaneous determination of target anesthetics, offering simplicity, high sensitivity, and specificity.

### 2.2. Selection of Developing Solvent

**Figure 2:**
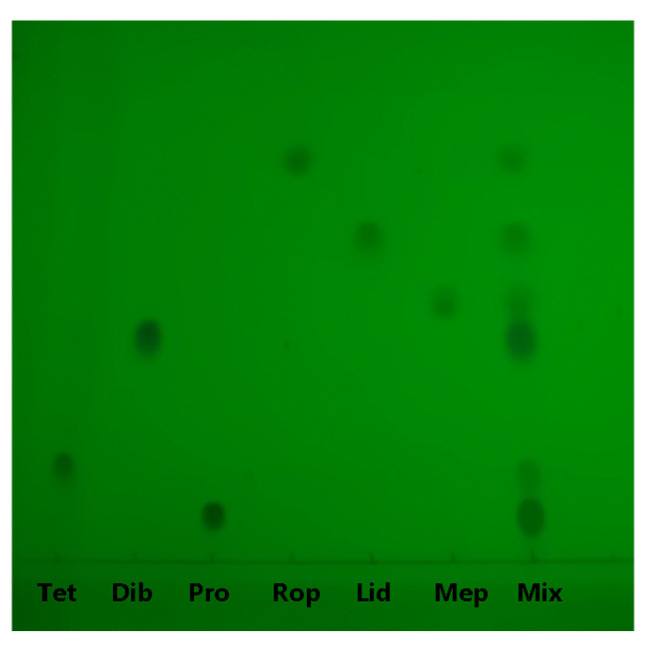
TLC results under a 254 nm ultraviolet lamp for the standard solution of each anesthetic and their standard mixture with the concentration of 2 mg/mL and 5 mg/mL. Developing system: cyclohexane–triethylamine system (13:7). The spotting volume is 2 μL. “Mix” means “the mixed standard solution”.

According to the report on the identification of anesthetics by TLC analysis and our previous results [24], the cyclohexane–triethylamine system was selected as the developing solvent for the effective separation of target compounds. We investigated the effect of the volume ratio of cyclohexane to triethylamine on separation efficiency, as shown in Figure S1. The target anesthetics were well separated using the cyclohexane–triethylamine system at a volume of 13:7 under our experimental conditions. It should be noted that the optimal developing solvent ratio may vary slightly with temperature and humidity, which can be addressed by making fine adjustments to the volume ratio. The TLC results of the standard solutions of each anesthetic and their mixed standard solution are shown in Figure 2. The mixed standard solution of local anesthetics was clearly separated due to differences in polarity, with *R*_f_ values of 0.08, 0.15, 0.40, 0.51, 0.60, and 0.73 for Pro, Tet, Dib, Mep, Lid, and Rop, respectively. These values were essentially identical to those of the individual standard solutions of each local anesthetic. This revealed that we achieved preliminary identification of the target compounds by TLC.

### 2.3. Selection of Ag NPs as SERS Enhancement Substrate

**Figure 3:**
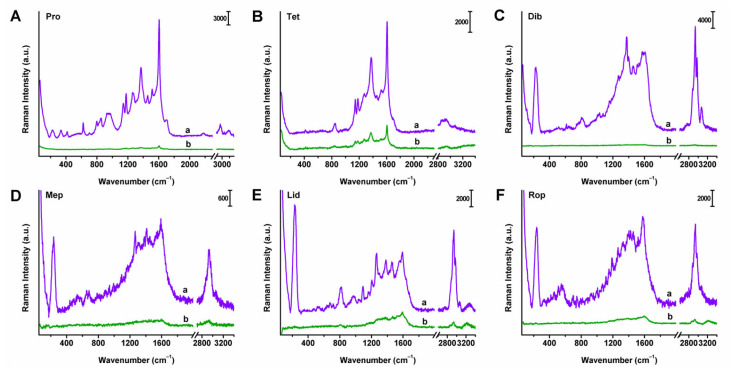
SERS spectra of concentrated TLC spots from 6 local anesthetics: (**A**) Pro, (**B**) Tet, (**C**) Dib, (**D**) Mep, (**E**) Lid, and (**F**) Rop, loaded with Ag NPs A (a) and B (b). Excitation wavelength: 780 nm.

Noble metal nanomaterials are widely recognized as traditional and effective substrates for SERS. Among them, Ag NPs generally exhibit stronger SERS enhancement ability than Au NPs. Ag NPs prepared by different methods show varying SERS enhancement responses. In our study, two types of Ag NPs were synthesized using the microwave heating method (marked as A) and a modified Lee and Meisel method (marked as B), respectively. The microwave method is easier to operate, requiring only a heating procedure of 6 min. UV-vis characterizations of Ag NPs A and B at the same dilution ratio are presented in Figure S2. The characteristic absorption peaks were located at 420 and 430 nm, respectively. Moreover, Ag NPs A exhibited stronger absorption intensity and narrower peak width than Ag NPs B due to a higher concentration of silver nanoparticles. The TEM images are shown in Figure S3, and the sizes of Ag NPs A and B are estimated to be 58.8 ± 8.8 and 62.3 ± 7.5 nm. The SERS enhancement abilities of Ag NPs A and B for concentrated TLC spots of six local anesthetics were particularly investigated in our study. As shown in Figure 3, Ag NPs A provided stronger and more abundant SERS signals for all anesthetics compared to Ag NPs B under the same experimental conditions at a 780 nm excitation wavelength. Herein, a 780 nm excitation light source was used to minimize fluorescence interference. Therefore, the microwave heating method was selected for synthesizing the SERS-active substrate for subsequent experiments due to its convenient operation and comparatively better SERS enhancement ability.

### 2.4. Raman and SERS Spectra

**Figure 4:**
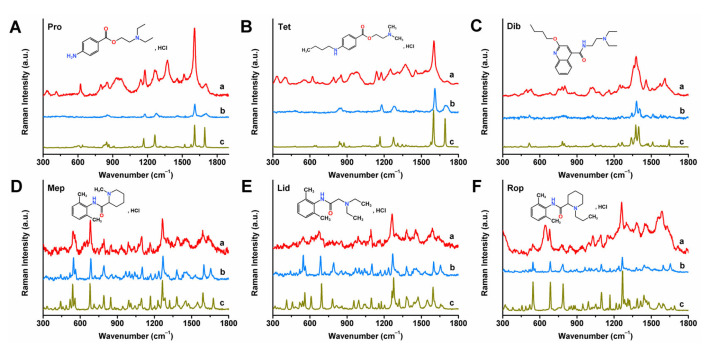
SERS (a) and Raman (b) spectra of concentrated TLC spots from 6 local anesthetics: (**A**) Pro, (**B**) Tet, (**C**) Dib, (**D**) Mep, (**E**) Lid, and (**F**) Rop. Raman spectra (c) of anesthetics solids. The spotting amounts for SERS spectra were 4 (Pro), 4 (Tet), 4 (Dib), 10 (Mep), 10 (Lid), and 10 (Rop) μg, respectively. Excitation wavelength: 780 nm.

Six local anesthetics were initially identified based on their spot positions matching those of the corresponding standard solutions (*R*_f_ values) using the TLC method. However, the specificity of TLC has certain limitations in sensitivity and selectivity, which cannot achieve precise identification. For example, two compounds with similar polarities may not be well separated by TLC. Additionally, no visible spots are formed at low concentrations. These limitations can be overcome by hyphenated techniques combined with Raman spectroscopy. Raman spectroscopy is a prevailing analysis technology owing to its molecular fingerprinting capability, non-destructive detection, and compatibility with aqueous samples. Moreover, advancements in plasmonics and instrumentation have enabled SERS technology to realize its potential for trace detection of biomolecules, disease diagnosis, and monitoring, primarily due to its significantly enhanced sensitivity down to the single-molecule level. Therefore, Raman and SERS characterizations (Figure 4) were performed on solvent-driven enriched TLC spots for the accurate identification of target local anesthetics. It was demonstrated that the Raman and SERS spectra of the enriched TLC spots show high consistency with the corresponding Raman spectra of pure solids, and each drug exhibits unique Raman and SERS responses, including the positions and shapes of characteristic peaks. The characteristic peaks and their assignments are deduced and summarized in Table S1 according to previous reports. The very strong SERS bands at 1603 (Pro) and 1603 (Tet) cm^−1^ were assigned to the ring C = C stretching vibrational mode [25]. For Mep and Rop, the SERS bands observed at 1266 and 1262 cm^−1^ corresponded to C-N stretching. The peak at 1264 cm^−1^ in the Lid SERS spectrum was assigned to CH bending and o-C-C stretching vibrations [26]. These results indicate that the proposed TLC-SERS strategy based on solvent-driven enrichment can enable exclusive identification of the six local anesthetics.

### 2.5. Spotting Amount-Dependent SERS Spectra

On the basis of its excellent discrimination performance, the sensitivity of our method was further investigated through a concentration gradient experiment. Standard solutions of a series of concentrations were spotted, enriched in situ under 254 nm UV light irradiation, and directly measured by SERS on the TLC plate. It is noteworthy that the sample spots gradually become invisible due to the decrease in concentration, which is detrimental to subsequent SERS analysis. In order to overcome this issue, a standard solution with a higher concentration was simultaneously spotted on the same TLC plate alongside analyte solutions at different concentrations to locate their spot positions for solvent-driven enrichment and Ag NPs staining. Images of the TLC plates before and after in situ concentration are shown in Figure S4.

**Figure 5:**
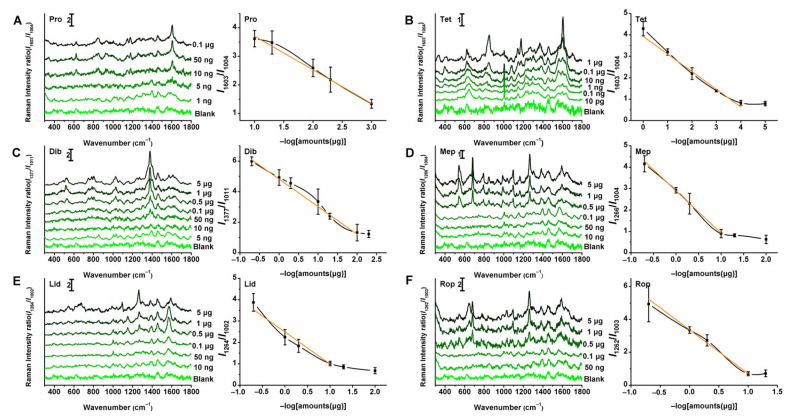
Representative spotting amount-dependent SERS spectra of 6 local anesthetics and the plot of SERS intensity ratio versus negative logarithm of spotting amounts sampled on TLC plates: (**A**) Pro, (**B**) Tet, (**C**) Dib, (**D**) Mep, (**E**) Lid, and (**F**) Rop. The yellow lines represens corresponding linear polynomial fitting results. Each error bar indicated the standard deviation of the SERS intensity ratio. The error bars were made with at least five sets of data.

SERS measurements were performed after Ag NPs staining on the enriched spots. All SERS spectra were processed and analyzed using Labspec 5 and OriginPro 8.5. We normalized the spectra at around 1005 cm^−1^ from the background SERS signal of the TLC plate. Representative spotting amount-dependent SERS spectra of the six local anesthetics are shown in Figure 5, exhibiting a trend in which the peak intensity ratio decreases with the reduction in sample spotting amounts. The very strong peaks at 1603 (Pro, Tet), 1377 (Dib), 1266 (Mep), 1264 (Lid), and 1262 (Rop) cm^−1^ were selected as characteristic bands for quantitative analysis. Linear correlations between the SERS intensity ratio of the characteristic bands and the negative logarithm of the spotting amounts were further investigated, and the corresponding plots are illustrated in Figure 5. The results demonstrated that the SERS intensity ratio of each anesthetic’s characteristic peak showed a strong linear relationship with the negative logarithm of the spotting amounts, as summarized in Table 1. Additionally, the lowest detectable amounts in this study were 1 ng, 10 pg, 10 ng, 50 ng, 50 ng, and 0.1 μg, with the sensitivity enhanced by up to 2 × 10^4^ times compared to that of our previous report using the TLC-Raman method [24]. These spotting amount-dependent SERS results demonstrate the potential of the proposed TLC-SERS method for trace detection of the six local anesthetics, attributed to the convenient separation capability of TLC, the solvent-driven enrichment approach, and the ultra-sensitivity and specificity of SERS.

### 2.6. Determination of Human Plasma Samples and Verification by HPLC

**Figure 6:**
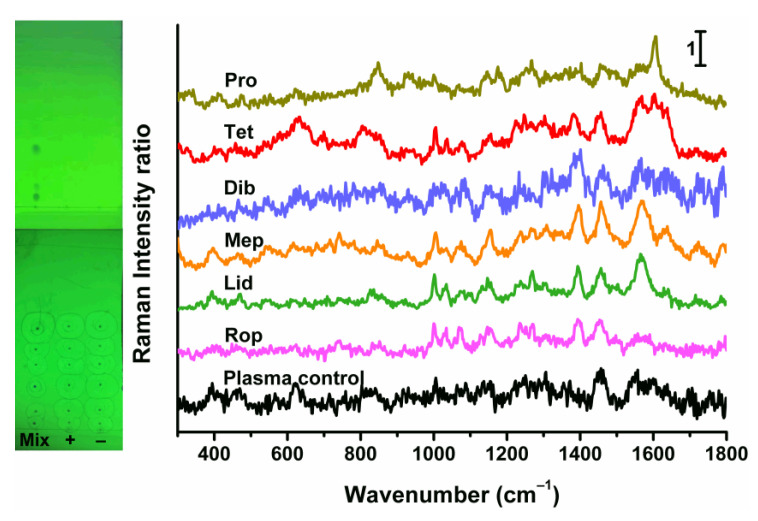
Image of TLC plate for human plasma samples and corresponding SERS responses of 6 local anesthetics in spiked samples. “Mix” means “the mixed standard solution”. “+” and “−“ represent positive and plasma control samples.

In order to investigate the feasibility of our method for real samples, human plasma samples were prepared using the standard addition method and tested according to our protocol. Plasma control, positive sample, and standard mixture were spotted on a TLC plate. The TLC images under 254 nm UV light irradiation before and after solvent-driven enrichment are shown in the inset of Figure 6. Although the TLC spots of the positive and control samples are invisible to the naked eyes, in situ concentration by solvent can be performed due to their identical *R*_f_ values with the standard solution. The SERS spectra of all analytes are illustrated in Figure 6. It can be clearly observed that no significant SERS information was detected for the plasma control spot, indicating that the human plasma matrix does not interfere with the multiplex SERS analysis of the local anesthetics we studied. Moreover, the SERS responses of the enriched spots from the positive plasma sample were highly consistent with those of the standard solution. The measured amounts of each anesthetic were calculated using the corresponding linear equations, thereby obtaining the recovery rates, calculated as the ratio of the measured amounts to the added amounts. As shown in Table 2, the recoveries for the target anesthetics in plasma samples ranged from 94.3% to 106.0%, with the RSD not exceeding 7.1% using this method. These results preliminarily confirm the accuracy and precision of the method. Furthermore, the concentrations of anesthetics in the human plasma positive samples were determined and validated by HPLC. The results obtained in this study agreed well with the corresponding HPLC data. Detailed HPLC methods and validation data are provided in the Supporting Information (Figure S5 and Tables S2 and S3). It was proved that the proposed TLC-SERS strategy based on solvent-driven enrichment is feasible and reliable for the multiplex determinations of local anesthetics in human plasma, with good accuracy and precision.

### 2.7. Stability

To evaluate the stability of the method, we spotted 8 parallel Tet spots with the spotting amounts of 1 μg. The plate was stored in a self-sealing bag in a dark place, and the SERS measurements were conducted under ambient conditions. Time-dependent SERS responses over 8 days were recorded, as shown in Figure S6. The variation in the SERS intensity ratio of Tet was not significant over this period, demonstrating the stability and practical applicability of this approach.

## 3. Materials and Methods

### 3.1. Chemical Reagents

Silver nitrate and sodium citrate were obtained from Sigma-Aldrich Co., Ltd. (Shanghai, China). Tetracaine hydrochloride (CAS 136-47-0), procaine hydrochloride (CAS 51-05-8), ropivacaine hydrochloride (CAS 132112-35-7), lidocaine hydrochloride (CAS 6108-05-0), mepivacaine hydrochloride (CAS 1722-62-9), and dibucaine (CAS 85-79-0) were purchased from Shanghai Jingchun Biochemical Technology Co., Ltd. (Shanghai, China). TLC silica gel 60F254 (SKU 1055540001) was purchased from Millipore Ltd. (Toronto, Canada). Carbamazepine (CAS 298-46-4) was obtained from Shanghai Aladdin Biochemical Technology Co., Ltd. (Shanghai, China). Cyclohexane and methanol were supplied by Fuchen (Tianjin) Chemical Reagent Co., Ltd. (Tianjin, China). Triethylamine was obtained from Tianjin Kemio Chemical Reagent Co., Ltd. (Tianjin, China). All these chemicals were analytical-grade reagents and used directly without further purification. The purified water purchased from Wahaha Group Co., Ltd. (Hangzhou, Zhejiang, China) was used throughout the experiment.

### 3.2. Apparatus and Measurement

Raman and SERS spectra were measured with the Thermo Scientific DXR3xi Raman Imaging Microscope (Waltham, MA, USA) equipped with an excitation laser wavelength of 780 nm (laser power: 24.0 mW) at room temperature. All Raman spectra were recorded by a 50× microscope objective. The laser power, exposure time, and scanning times of target anesthetics were different and listed in Table S4. The pinhole was 25 μm. The obtained Raman spectra were baseline corrected by NGS LabSpec 5 and plotted with OriginPro 8.5. Other instruments included BYLAB three by ultraviolet analyzer from Beijing Bingyang Technology Co., Ltd. (Beijing, China) equipped with 254 and 365 nm light sources, CNC ultrasonic cleaner KQ-500DE from Kunshan Ultrasonic Instrument Co., Ltd. (Kunshan, Jiangsu, China), high speed centrifuge TG16 from Shanghai Huxiangyi Centrifuge Instrument Co., Ltd. (Shanghai, China), vortex mixer from Qun’an Instrument Co., Ltd. (Huzhou, Zhejiang, China), 2 μL quantitative spotting capillary from Tianjin Enlida Technology Co., Ltd. (Tianjin, China), and chromatographic tank from Shanghai Huake Laboratory Equipment Co., Ltd. (Shanghai, China). Transmission electron microscopy (TEM) images were taken on a Hitachi-HT7700 transmission electron microscope (Hitachi, Tokyo, Japan) at an accelerating voltage of 200 kV. The HPLC system was LC-1000 from Shandong Lunan Ruihong Chemical Instrument Co., Ltd. (Lunan, Shandong, China) equipped with a sample injection pump, an automatic sampler, and an ultraviolet (UV) detector. For detailed HPLC conditions, refer to the Supporting Information.

### 3.3. Preparation of Ag NPs

Ag NPs A were prepared by the microwave heating method. Silver nitrate (100 mL, 2 × 10^−3^ mol/L) was reduced by sodium citrate (2.6 mL, 1%) under microwave heating for 6 min. Ag NPs B were synthesized by the modified Lee and Meisel method [27]. Silver nitrate (200 mL, 10^−3^ mol/L) in a slightly boiling state was reduced by sodium citrate (4 mL, 1%), and the solution was kept at 85~90 °C for 40 min under magnetic stirring. The produced Ag NPs were ready for use after cooling to room temperature.

### 3.4. Preparation of Standard Solutions

Local anesthetics were precisely weighed and dissolved in methanol, obtaining the standard stock solution with the concentration of 2, 2, 2, 5, 5, and 5 mg/mL for Pro, Tet, Dib, Mep, Lid, and Rop, respectively. Standard solutions in different concentrations were obtained by the stepwise dilution method. Mixed standard solutions were also diluted with six stock standard solutions by methanol for TLC analysis.

### 3.5. Preparation of Human Plasma Samples

Human plasma was provided by the Second Affiliated Hospital of Qiqihar Medical University. Briefly, the blood samples in the vacuette blood collection tubes were centrifuged at 3500 rpm for 10 min, and the upper layer was the plasma layer. The mixture of 5 mL plasma and 15 mL methanol was vortexed for 1 min, ultrasonically treated for 5 min, and then centrifuged at 12,000 rpm for 10 min. We used the supernatant as a plasma control. The positive plasma samples, including 6 anesthetics for TLC-SERS analysis, were prepared by diluting the mixing stock solutions with plasma control. The final concentrations of Pro, Tet, Dib, Mep, Lid, and Rop were 3, 3, 8, 60, 60, and 60 μg/mL. Then, standard stock solutions of each analyte (1 mg/mL) and carbamazepine (Car, internal standard, 1 mg/mL) were prepared with methanol. A series of working solutions of each analyte in different concentrations (100, 50, 25, 10, 5, 1 μg/mL) for HPLC analysis were prepared by diluting the stock solutions with plasma control. The final concentration of Car in working solutions was 50 μg/mL. Plasma samples were freshly prepared before use.

### 3.6. TLC Separation, Solvent-Driven Enrichment, and SERS Measurement for Anesthetics

Two microliters of the solutions to be measured were spotted on the silica gel thin-layer plate using a quantitative capillary. After the sample spots had dried naturally, the spotted thin-layer plate was pre-saturated for 15 min with 5 mL cyclohexane–triethylamine (*v*:*v* = 13:7) solution as the developing solvent in the chromatographic tank. After the developing solvent was fully expanded, the locations of sample spots were marked using a pencil under 254 nm UV light irradiation, and the *R*_f_ values were then calculated.

Then, the sample spots were enriched to much smaller points through the promotion of solvent (methanol) with spotting quantitative capillary from different directions. The enriched TLC spots were cut from the whole plate, obtaining a square aluminum plate loaded with anesthetics. The plate was placed in the overturned cap of a 1.5 mL EP tube. After that, 10 μL of Ag NPs were added into the cap to make sure that the square aluminum plate is completely immersed in Ag NPs. SERS measurements were conducted on a square aluminum plate in a slightly wet state with 780 nm excitation wavelength. The sampling volumes were 2 μL for all samples.

## 4. Conclusions

In this work, we developed a TLC-SERS approach for solvent-driven enrichment and multiplex determination of local anesthetics. The TLC spot of each anesthetic was concentrated into a smaller area through solvent-driven enrichment. SERS fingerprint signals were then collected from the concentrated TLC spots using Ag NPs colloid staining, which significantly enhanced the sensitivity and selectivity. Human plasma samples were analyzed by the TLC-SERS method with simple sample pretreatment, demonstrating good accuracy and precision. This study provides a simple, selective, and sensitive approach for the multiplex analysis of local anesthetics in biological fluids, which can largely enhance the safe and effective use of local anesthetics.

## Figures and Tables

**Table 1:** Summary of results of the calibration curves for determining local anesthetics by the proposed TLC-SERS method.

Analytes	Linear Equation	Linear Range	R^2^	Lowest Detectable Amounts	Lowest Detectable Amounts by TLC-Raman [24]
Pro	y = 4.886139 − 1.178272x	0.1 μg–1 ng	0.9961	1 ng	0.4 μg
Tet	y = 3.93642 − 0.824827x	1 μg–0.1 ng	0.9742	10 pg	0.2 μg
Dib	y = 4.828164 − 1.806599x	5 μg–10 ng	0.9920	10 ng	1.2 μg
Mep	y = 2.897006 − 1.975927x	5–0.1 μg	0.9984	50 ng	1 μg
Lid	y = 2.484772 − 1.492415x	5–0.1 μg	0.9714	50 ng	1 μg
Rop	y = 3.369356 − 2.664862x	5–0.1 μg	0.9981	0.1 μg	1 μg

**Table 2:** Recovery results for human plasma samples by the TLC-SERS method.

Analytes	Added Amounts (ng)	Measured Amounts (ng)	Recovery (%)	RSD (%)
Pro	6	6.12 ± 0.33	102.1	5.5
Tet	6	5.87 ± 0.38	97.9	6.5
Dib	16	15.75 ± 0.83	98.4	5.3
Mep	120	122.15 ± 5.28	101.8	4.4
Lid	120	127.21 ± 9.03	106.0	7.1
Rop	120	113.21 ± 6.51	94.3	5.8

## Data Availability

The original contributions presented in this study are included in the article/Supplementary Material. Further inquiries can be directed to the corresponding author(s).

## Supplementary Materials

The following supporting information can be downloaded at https://www.mdpi.com/article/10.3390/molecules30071585/s1. Figure S1: TLC plates for separation of standard anesthetics with cyclohexane–triethylamine system in different volume ratios; Figure S2: UV-Vis characterizations of Ag NPs A and B; Figure S3: TEM characterization and size distribution of Ag NPs; Figure S4: The TLC plates of standard anesthetics in a series of concentrations before and after in situ enrichment; Figure S5: HPLC chromatogram of the positive plasma sample; Figure S6: Stability test; Table S1: Characteristic Raman, SERS bands and their assignments of local anesthetics; Table S2: Calibration curves for anesthetics by HPLC internal standard method; Table S3: Comparison between TLC-SERS and HPLC method for positive plasma sample; Table S4: SERS measurement conditions.
