# Supplementary material for: Solvent-Driven Enrichment and Multiplex Analysis of Local Anesthetics by Thin-Layer Chromatography Coupled with Surface-Enhanced Raman Spectroscopy
Source: Molecules. 2025 Apr 2;30(7):1585. doi: 10.3390/molecules30071585 (PMC11990880; doi:10.3390/molecules30071585)
Supplement: Supplementary file 1 [file molecules-30-01585-s001.zip › molecules-3500123-supplementary.pdf]

***Supporting Information for Publication***

# Solvent-Driven Enrichment and Multiplex Analysis of Local Anesthetics by Thin-Layer Chromatography Coupled with Surface-Enhanced Raman Spectroscopy

**Huimin Sui [1,2], Miao Li [1], Yangyang Gao [1], Jie Luo [1], Fangyuan Ban [1], Tao Xu [1], Shuang Fu [1], Chao-Yang Zhao [1], Hailin Wen [1] and Cuiyan Han [1,2,*]**

[1] School of Pharmacy, Qiqihar Medical University, Qiqihar 161006, China; suihm_9@163.com (H.S.); 19190706959@163.com (M.L.); 13283631961@163.com (Y.G.); 18074757884@163.com (J.L.); 18522488569@163.com (F.B.); harvey-333@163.com (T.X.); fsjt1980@163.com (S.F.); zhao33447@qmu.edu.cn (C.-Y.Z.); hlw395456@163.com (H.W.)

[2] Postdoctoral Research Station, Qiqihar Institute of Medical and Pharmaceutical Sciences, Qiqihar 161006, China

* Correspondence: hcymuphar@qmu.edu.cn

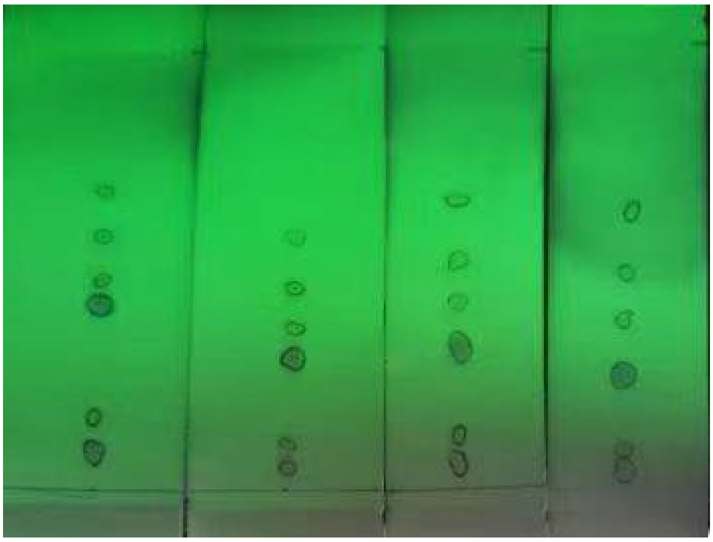

**Figure S1.** TLC plates for separation of standard anesthetics with cyclohexane-triethylamine system in different volume ratios (3:1; 7:3; 13:7; 6:4).

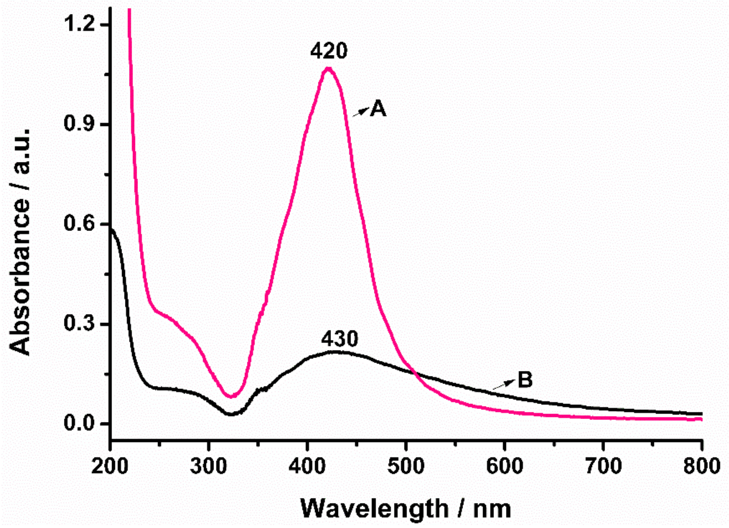


**Figure S2.** UV-vis characterizations of Ag NPs A and B.

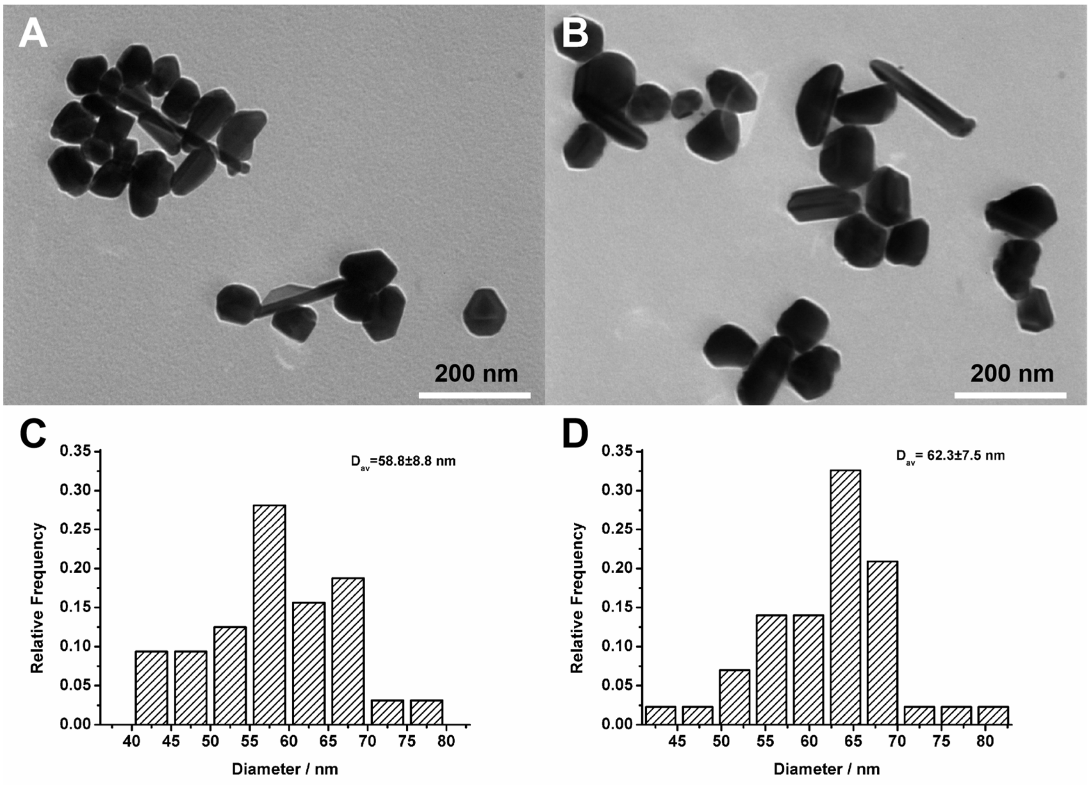


**Figure S3.** TEM characterization and size distribution of Ag NPs A (A, C) and B (B, D).

**Table S1.** Characteristic Raman, SERS bands and their assignments of local anesthetics.

| Name | Structure | Raman ($cm^{-1}$) | SERS ($cm^{-1}$) | Raman for solid ($cm^{-1}$) | Assignment |
|---|---|---|---|---|---|
| Pro | 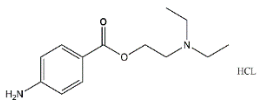 | 3076 | | 3210、3075 | $\nu(NH_2)$ |
| | | 2984、2949 | 2931 | 2985、2943 | $\nu(CH_3)$ |
| | | 1704、1691 | | 1695 | $\nu(C=O)$ |
| | | | | 1645 | $\beta(NH_2)$ |
| | | 1608 | 1603 | 1606 | $\nu(C=C)$ |
| | | | | 1263 | $\nu(CO)$ |
| | | 1176 | 1172 | 1165 | $\gamma(C-C-N-H)$ & $\gamma(C-H-C-C)$ |
| | | 856 | 853 | 847 | $\nu(C-C)$ & $\nu(O-C)$ |
| Tet | 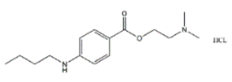 | | | 3377 | $\nu(NH_2)$ |
| | | 2972、2964 | | 2966、2952 | $\nu(CH_3)$ |
| | | 1697 | 1709 | 1693 | $\nu(C=O)$ |
| | | 1609 | 1601 | 1599 | $\nu(C=C)$ |
| | | 1279 | 1249 | 1275 | $\nu(CO)$ |
| | | 1179 | 1172 | 1167 | $\gamma(C-C-N-H)$ & $\gamma(C-H-C-C)$ |
| | | 839 | 851 | 839 | $\nu(C-C)$ & $\nu(O-C)$ |
| Dib | 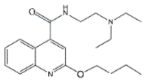 | 3072 | | 3078、3066 | $\nu(NH)$ |
| | | | | 1645 | $\nu(C=O)$ |
| Mep | 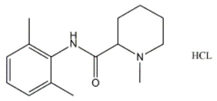 | 1655 | 1627 | 1676 | $\nu(C=O)$ |
| | | 1599 | 1590 | 1593 | $\beta(NH)$ |
| | | 1440、1381 | 1453、1384 | 1454、1381 | aromatic ring skeleton |
| | | 1269 | 1264 | 1265 | $\nu(C-N)$ |
| | | 1100 | 1096 | 1095 | $\nu(C_{ring}-N)$ |
| | | 544 | 541 | 538 | $\nu(O=C-C)$ |
| Lid | 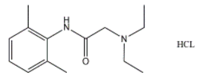 | 2927 | | 2925 | $\nu(CH_2)$ |
| | | 1597 | 1590 | 1595 | $\delta(HNC)$ & $\nu(NC)$ |
| | | | 1455 | 1473 | $\delta(CH_2)$ & $\beta(CH)$ |
| | | | 1382 | 1381 | $\omega(CH_2)$ |
| | | 1267 | 1264 | 1263 | $\beta(CH)$ & $\nu(o-C-C)$ |
| | | 1098 | 1092 | 1095 | $\rho(CH_3)$ & $\tau(CH_2)$ |
| | | 982 | 990 | 993 | $\rho(CH_2)$ & $\omega(CH)$ |
| | | | 672 | 694 | $\omega(HNC)$ & $\tau(HNC)$ & $\delta_{ring}$ |
| Rop | 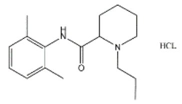 | 1654 | 1628 | 1687、1660 | $\nu(C=O)$ |
| | | 1597 | 1590 | 1604、1595 | $\beta(NH)$ |
| | | 1444、1380 | 1450、1382 | 1440、1386 | aromatic ring skeleton |
| | | 1266 | 1260 | 1269 | $\nu(C-N)$ |
| | | 1097 | 1094 | 1097 | $\nu(C_{ring}-N)$ |
| | | 544 | 543 | 544 | $\nu(O=C-C)$ |

*ν*-stretching, *β*-in-plane bending, *δ*-deformation, *γ*-outof-plane bending, *ρ*-rocking, *τ*-torsion, *ω*-wagging.

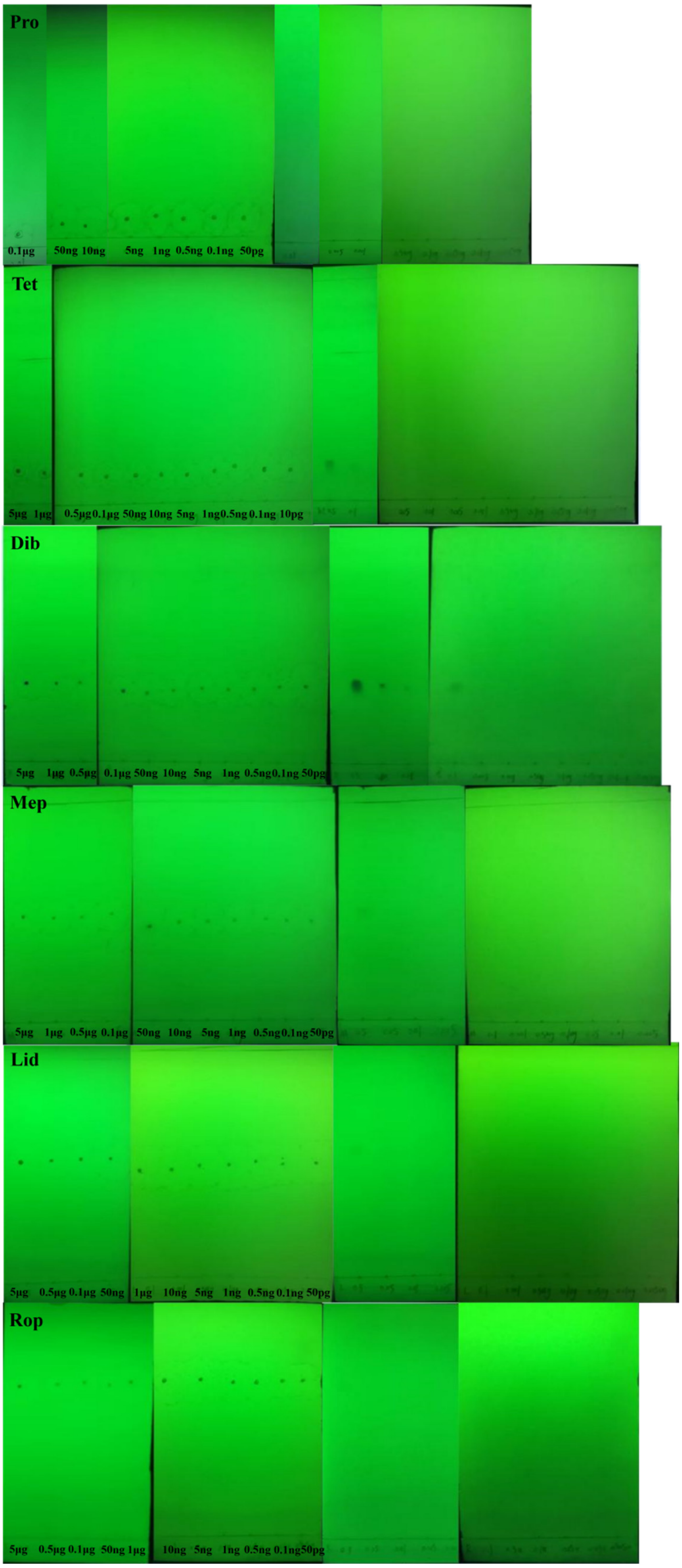


**Figure S4.** The TLC plates of standard anesthetics in a series of concentrations before and after in situ enrichment. The amounts of spots on TLC plates: Pro(0.1 μg-50 pg); Tet(5 μg-10 pg); Dib(5 μg-50 pg); Mep(5 μg-50 pg); Lid(5 μg-50 pg); Rop(5 μg-50 pg).

**HPLC Method**

The HPLC system was LC-1000 (Shandong Lunan Ruihong Chemical Instrument Co., Ltd, China) equipped with a sample injection pumb, an automatic sampler, an ultraviolet (UV) detector. The HPLC setting conditions were slightly modified with reference to the 《Methods for the determination of 7 substances including lidocaine in cosmetics》 (GGTG-2015-12006) announced on September 28, 2015 by National Medical Products Administration for this experiment. Chromatographic separation of the anesthetics was completed on a Kromasil-C18 reversed-phase column (4.6 mm×250 mm, 5 μm), and the mobile phase consisted of a 0.01 mol/L $Na_2HPO_4$ aqueous solution (adjust pH to 7.0 with $H_3PO_4$, solvent A) and methanol (solvent B), with the following conditions: 0-6 min, 60%B; 6-16 min, 60%B; 16-20 min, 60%-80%B; 20-40 min, 80%B. The instrument parameters were set to a run time of 40 min, an injection vlume of 20 μL, an eluent flow rate of 1.0 mL/min, and a column temperature of 25℃. An UV spectrophotometer was used to detect the characteristic detection wavelengths of the anesthetics. The UV detection wavelength was 230 nm for all the six anesthetics. HPLC standard solutions were prepared by adding standard solution of each anesthetic in different volumes into plasma control, making the final concentrations 1, 5, 10, 25, 50 and 100 μg $mL^{-1}$. At the same time, each standard solution contained carbamazepine (Car, 50 μg $mL^{-1}$) as an internal standard. The samples were filtered by 0.22 μm filter membrane before HPLC measurements.

**HPLC results**

As shown in **Figure S5**, the six anesthetics in the positive plasma sample were well separated by HPLC method, and the retention time was 8.9, 11.2, 14.6 , 25.1 , 27.8 , 26.3 and 33.1 min for Pro, Car, Mep, Lid, Tet, Rop and Dib, respectively. We calculated the linear equation of peak area ratio versus concentration for each anesthetic by internal standard method, the results of the calibration curves for determining local anesthetics by HPLC method were listed in **Table S2**. Additionally, the comparison between HPLC and TLC-SERS method in terms of positive plasma sample was shown in **Table S3**.

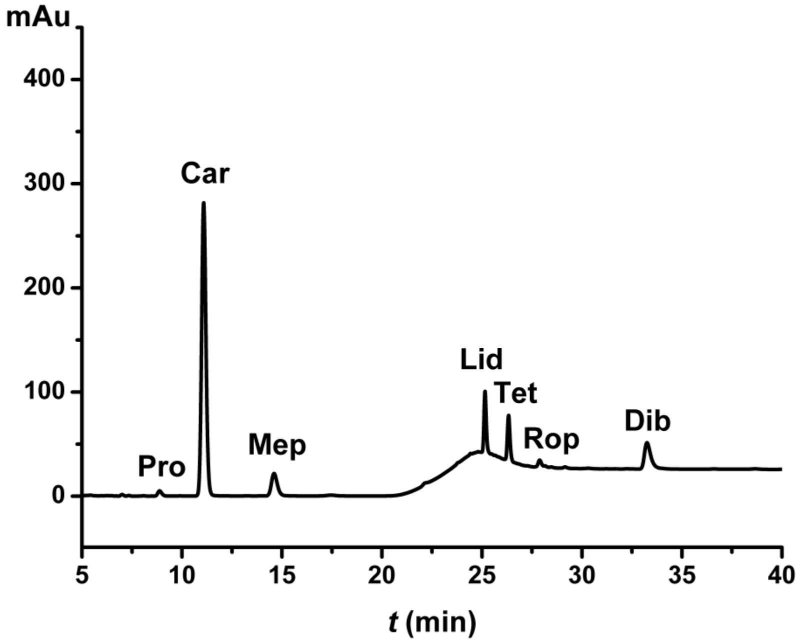


**Figure S5.** HPLC chromatogram of the positive plasma sample with the concentration of 3, 3, 8, 60, 60, 60 and 50 μg/mL for Pro, Tet, Dib, Mep, Lid, Rop and Car, respectively.

**Table S2.** Summary of results of the calibration curves for determining local anesthetics by the HPLC internal standard method.

| Analytes | Linear equation | Linear range (μg/mL) | $R^2$ |
|---|---|---|---|
| Pro | y = 0.0055x - 0.0011 | 1-100 | 0.9962 |
| Tet | y = 0.005x + 0.0287 | 1-100 | 0.9958 |
| Dib | y = 0.018x + 0.065 | 1-100 | 0.9959 |
| Mep | y = 0.0039x + 0.0042 | 1-100 | 0.9967 |
| Lid | y = 0.0039x + 0.0132 | 1-100 | 0.9995 |
| Rop | y = 0.0031x + 0.0037 | 1-100 | 0.9984 |

**Table S3.** Comparison results between TLC-SERS and HPLC method in terms of positive plasma sample.

| Analytes | Added amounts (ng) | HPLC (ng) | TLC-SERS method (ng) |
|---|---|---|---|
| Pro | 6 | 6.05 | 6.12±0.33 |
| Tet | 6 | 5.93 | 5.87±0.38 |
| Dib | 16 | 16.17 | 15.75±0.83 |
| Mep | 120 | 123.78 | 122.15±5.28 |
| Lid | 120 | 116.02 | 127.21±9.03 |
| Rop | 120 | 116.16 | 113.21±6.51 |

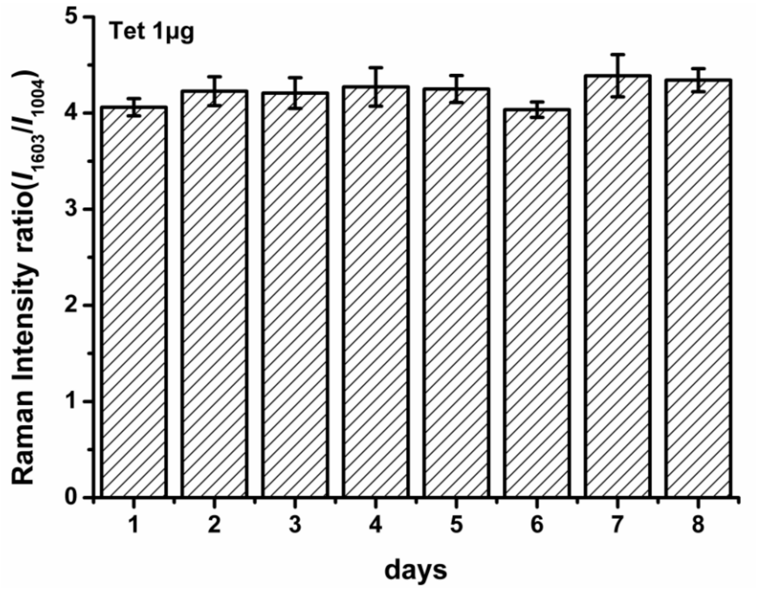


**Figure S6.** SERS intensity ratio of enriched Tet (1 μg) TLC spot in 8 days.

**Table S4.** Measurement conditions of SERS.

| Analytes | Laser power (mW) | Exposure time (s) | Scanning times |
|---|---|---|---|
| Tet | 10.0 | 1.00 | 15 |
| Dib | 10.0 | 5.00 | 20 |
| Pro | 10.0 | 0.05 | 20 |
| Rop | 10.0 | 0.25 | 20 |
| Lid | 10.0 | 0.50 | 20 |
| Mep | 10.0 | 2.00 | 20 |
